# The Impact of Self‐Directed Aftercare Following Breast Cancer Surgery: A Scoping Review

**DOI:** 10.1002/pon.70368

**Published:** 2025-12-29

**Authors:** Anna T. Isaac, Olinda Santin, Gareth W. Irwin, Katherine Mankelow, Louise V. Cousins, Stuart A. McIntosh

**Affiliations:** ^1^ Johnston Centre for Cancer Research Queen's University Belfast Belfast UK; ^2^ Department of Breast Surgery Belfast City Hospital Belfast UK; ^3^ School of Nursing and Midwifery Queen's University Belfast Belfast UK

## Abstract

**Purpose:**

With over 56,000 new cases of breast cancer a year in the UK and 76% of these expected to live beyond 10 years, managing long‐term care and support is an urgent challenge. This scoping review aims to map the current literature on outcomes and lived experience of Self‐Directed Aftercare (SDA) pathways following breast cancer surgery. We aim to assess what evidence exists to support the current delivery of this approach.

**Methods:**

A scoping review in line with the PRISMA‐ScR template was undertaken across 3 databases (Web of Science, PubMED and OVID Medline) using an iteratively developed search strategy based on concepts of “breast cancer”, “self‐directed” and “aftercare”. Screening was undertaken by all authors and disagreements settled by team discussion. Key data were extracted from qualitative and quantitative studies, with descriptive statistical and thematic analysis conducted.

**Conclusions:**

Available literature is sparse and of variable quality. While reductions in clinic attendance are reported, there is a wide range of patient lived experience. There are positive reports of the convenience of SDA, while negative aspects included unmet psychological and information needs, which also changed over time. Ease of access to speciality breast advice varied across studies.

**Implications for Cancer Survivors:**

Globally more patients are being managed via SDA, but this review demonstrates the lack of research assessing the safety, acceptability, and cost‐effectiveness of this approach. It is imperative that services address the evolving needs of breast cancer survivors and integrate feedback from patients with lived experience of breast cancer aftercare.

**Trail Registration:**

This project has been publicly registered with the Open Science Framework (July 2024). It can be found under the project “Exploring oncological outcomes and lived experiences of patients and their carers managed via self‐directed aftercare pathways following breast cancer surgery” available at https://osf.io/b56vq/.

## 1 Introduction

Breast Cancer is the most common cancer in women worldwide, with around 56,800 new cases diagnosed each year in the UK alone [1]. Breast cancer mortality continues to improve worldwide, with over 75% of women expected to survive for 10 or more years following treatment [1]. In light of both the increased incidence of breast cancer diagnoses and the fact that women are living longer post‐treatment, delivering a safe and cost‐effective aftercare service is a priority, particularly within resource‐limited settings. Simultaneously, patient feedback highlights that there are ongoing unmet needs in how aftercare is currently delivered [2]. There is a growing interest in how we can re‐imagine breast cancer aftercare to deliver safe follow‐up that is acceptable to patients while remaining cost‐effective.

Breast cancer follow up programmes aim to detect recurrent disease, facilitate ongoing psychological support, and identify and manage side‐effects or physical complications of treatment [3, 4, 5, 6]. Most involve regular mammography to detect local recurrence within the treated breast or a new cancer in either breast. A previous meta‐analysis concluded that clinical detection of recurrence was associated with less favourable outcomes than mammographic detection of asymptomatic disease, and that early detection of such in‐breast recurrence may increase available treatment options for patients [5]. In contrast, a retrospective study from the Netherlands suggested that routine clinical examination had no impact either on the detection of locoregional recurrence, nor on overall survival following this [7]. Many authors have suggested that asymptomatic patients do not merit routine clinical examination [7, 8, 9, 10].

It is well recognised that physician‐led regular follow‐up does not adequately address the holistic needs of breast cancer patients. Multiple reviews identify under‐reporting of symptoms by patients during their regularly scheduled review, and little to no dedicated time for exploring or addressing psychological needs [10, 11, 12, 13]. For some patients these regular reviews can also cause heightened and unnecessary anxiety, compounding other unmet psychological needs [10].

In the UK, as early as 2002, the National Institute for Clinical Excellence (NICE) recommended that ‘long‐term routine hospital‐based follow‐up [for breast cancer] should cease’ [14], and commented that ‘scarce resources are still being used for this largely ineffective activity’ [14]. This has contributed to the development of Self‐Directed Aftercare (SDA) programmes in breast cancer, with symptomatic patient‐initiated contact replacing routine outpatient reviews. Within SDA pathways, patients are still invited to regularly scheduled mammograms for a number of years post‐treatment, but they will not be seen by a healthcare professional for routine clinical review.

Despite growing interest in and uptake of this approach, there is relatively little known regarding its outcomes, acceptability to patients or staff, or even resource consumption. There is therefore an urgent need to understand whether such programmes best serve the needs of breast cancer patients and healthcare systems.

### 1.1 Objective

The objective of this scoping review was to assess evidence available regarding patient‐led approaches to aftercare following surgery for early breast cancer, to establish whether this approach has been shown to be safe, well tolerated by patients and carers, and cost‐effective.

## 2 Methods

A preliminary search of the Cochrane Library and JBI Evidence Synthesis did not identify any ongoing or planned scoping reviews on the topic. This review was carried out according to the recommendations of the Preferred Reporting Items for Systematic Reviews and Meta‐Analyses Extension for Scoping Reviews (PRSIMA‐ScR). A scoping review was favoured as this technique is helpful for mapping key concepts and identifying common terminology in areas which have not been extensively reviewed. Prior to conducting the search, the key concept of Self‐Directed Aftercare (SDA) was defined. To be considered an SDA pathway, publications needed to describe a follow‐up system where patients initiated contact with a healthcare professional rather than being regularly recalled (other than surveillance mammography). Papers were excluded if they involved a scheduled contact from a healthcare professional. Articles published since the year 2000 in/translated to English were included, as SDA was not widely practiced prior to this.

The following search strategy terms and Boolean operators were agreed upon: *‘breast cancer’* AND (*‘self‐directed’* OR *‘patient‐initiated’) AND (‘aftercare”* OR *‘follow‐up’*). Three databases (Web of Science, PubMed and Ovid MEDLINE) were searched. After a preliminary search, these terms were iteratively expanded to include: *“breast cancer’* AND (*‘self‐directed’* OR *‘patient‐directed’* OR *‘self‐led’* OR *‘patient‐led’* OR *‘self‐initiated’* OR *‘patient‐initiated’* OR *‘open access’*) AND (*‘aftercare’* OR *‘follow‐up’*) and the search was run again in the above three databases by AI, LC and KM (full search results in Supporting Information S1: Appendix 1). Initial duplication screening was undertaken using Covidence software [15] and abstract screening was carried out by AI, LC and KM. The search parameters were developed iteratively by AI, SMcI and OS. Pathways which expected patients to contact their own GP were excluded as this was not felt to reflect hospital‐led Self‐Directed Aftercare.

During abstract review, it was decided by all authors that conference proceedings, abstracts and review articles were excluded from full text screening. Following abstract screening, full text screening was undertaken and relevant articles retained. After agreement between all authors, publications considering multiple cancer sites were included if SDA in breast cancer was reported separately within the article. If data on cancers were merged, papers were excluded. Cited references for included papers were reviewed by AI to screen for additional relevant studies. An attempt was made to contact authors of one further paper where it was unclear if the population had been undergoing SDA or routinely scheduled follow‐up; no response was received and so this paper was excluded from analysis.

Data were extracted using an iteratively developed data extraction tool in Microsoft Excel (version 16.76) spreadsheet for presentation and analysis of data. Data points collected were: study characteristics (year and country of publication, author(s), study design, primary endpoints, sample size, cancer stage, patient age and sex), outcome measures, mechanism of follow‐up (details of self‐directed aftercare pathway), common terminology used to refer to Self‐Directed Aftercare, results, and interview/free text quotes from patients. Data was primarily collected by AI with cross‐checking of data performed by LC and KM.

Thematic analysis was conducted according to the six‐step method described by Braun & Clark [16]: familiarisation with data, generation of initial codes, generation of themes, review of themes, defining and naming themes, and write‐up. Coding and themes were developed between AI and OS. Where there was disagreement regarding naming of themes this was addressed by consultation with SMcI.

## 3 Results

**FIGURE 1:**
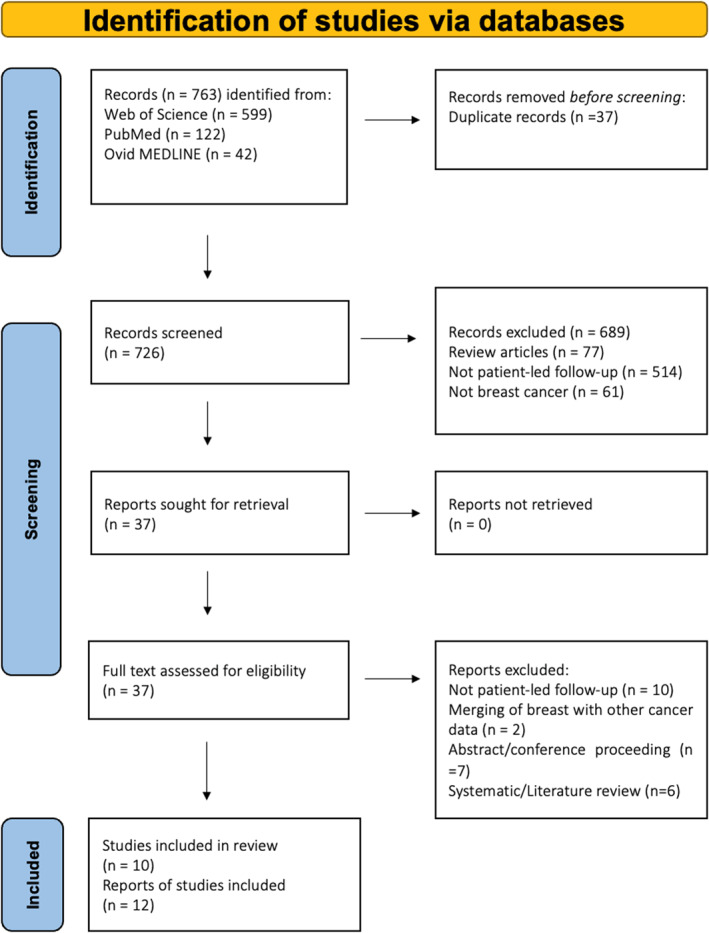
PRISMA flow‐chart of search strategy.

Figure 1 displays the PRISMA flow‐chart for the search strategy. 37 full text papers were screened and 12 were included in the final review, representing 10 original studies. The characteristics of each study are given in Tables 1, 2, 3, 4.

**TABLE 1:** Characteristics of studies.

	Study design	Primary endpoint	Sample size	Populations		
				Stage	Sex	Age
Brown et al. (2002) UK [17]	Randomised control trial (RCT)	• Quality of life (QoL) • Psychological morbidity • Satisfaction with follow‐up	61	I	Female	48–87
Capelan et al. (2017) UK [18]	Service evaluation	• Prevalence of unmet needs	625	I‐III	Female	30–97
Chapman et al. (2009) UK [19]	Audit	• Patient experience • Effect on GP workload	130	Nottingham prognostic index (NPI) < 4.4 or ductal carcinoma in situ (DCIS)	Male and female	Not given
Jenkins et al. (2023) UK [20]	Prospective observational	• QoL • Psychological morbidity • Service use	110	‘Early‐stage’	Male and female	Not given
Karlsen et al. (2023) Denmark [21]	Prospective observational	• Health related QoL (HRQoL)	987	I‐II	Female	> 40
Kirshbaum et al. (2016) UK [22]	RCT	• QoL	112	I‐II	Female	29–86
Koinberg et al. (2004) Sweden [23]	RCT	• Satisfaction with follow‐up	264	I‐II	Female	40–82
Moore et al. (2022) UK [24]	Observational	• Patient experience	118	I‐III	Female	Not given
Sheppard et al. (2009) UK [25]	RCT	• Psychological morbidity • QoL	237	I‐III	Female	Mean age 57
Riis et al. (2020) Denmark [26]	RCT	• Satisfaction with follow‐up	134	All stages	Female	Post‐menopausal only

**TABLE 2:** Outcome measures.

	Outcome measures													
	QLQ‐BR23	HADS	QLQ‐C30	FACT ‐B	EQ 5D‐5L	GSE	GHQ‐12	PRRS	Cost diary	Service use	Questionnaires	HNA	Interviews	Recurrence
Brown et al. (2002) UK [17]	✔	✔	✔							✔			✔	✔
Capelan et al. (2017) UK [18]												✔		
Chapman et al. (2009) UK [19]										✔	✔			
Jenkins et al. (2023) UK [20]			✔	✔	✔	✔	✔	✔	✔	✔			✔	
Karlsen et al. (2023) Denmark [21]	✔	✔	✔			✔				✔	✔	✔	✔	
Kirshbaum et al. (2016) UK [22]	✔	✔	✔											
Koinberg et al. (2004) Sweden [23]		✔								✔				✔
Moore et al. (2022) UK [24]										✔	✔			
Sheppard et al. (2009) UK [25]				✔			✔							✔
Riis et al. (2020) Denmark [26]	✔		✔							✔	✔			

Abbreviations: EQ5L‐DL = EUROQoL 5 dimensions 5 levels questionnaire [29, 30]; FACT‐B = functional assessment of cancer therapy ‐ breast [31]; GHQ‐12 = general health questionnaire [33]; GSE = general self‐efficacy scale [27]; HADS = hospital anxiety and depression scale [28]; HNA = holistic needs assessment [32]; PRRS = *unvalidated* patient roles and responsibilities score [20]; QLQ‐BR23 = quality of life questionnaire‐breast cancer [6]; QLQ‐30 = quality of life questionnaire [6].

**TABLE 3:** Mechanism of follow‐up.

	Mechanism of follow‐up						
	Mammogram	Nurse meeting	Group meeting	Written info	GP letter	ePROMs	Contact
Brown et al. (2002) UK [17]	Annual			✔			Telephone BCN
Capelan et al. (2017) UK [18]	Annual	✔		✔	✔	✔	Telephone BCN
Chapman et al. (2009) UK [19]	BCS = annual Mastectomy = 2 yearly	✔		✔	✔		Telephone BCN
Jenkins et al. (2023) UK [20]	Annual	✔		✔			Telephone BCN
Karlsen et al. (2023) Denmark [21]	Not specified	Not specified	Not specified	Not specified	Not specified	Not specified	Not specified
Kirshbaum et al. (2016) UK [22]	Annual for 5 years	✔	✔	✔	✔		Telephone BCN
Koinberg et al. (2004) Sweden [23]	Annual for 3 years	✔					Nurse or physiotherapist
Moore et al. (2022) UK [24]	Not specified	Not specified	Not specified	Not specified	Not specified	Not specified	Not specified
Sheppard et al. (2009) UK [25]	Annual	✔		✔			Telephone BCN
Riis et al. (2020) Denmark [26]	Annual	✔			✔		Telephone BCN

Abbreviation: ePROMS = electronic patient reported outcome measures.

**TABLE 4:** Common terminology.

	Key terms								
	Patient‐initiated follow‐up	Open access	Supported self‐ management	Patient‐led follow‐up	Nurse‐led follow‐up on demand	Specialist nurse‐led check‐up	Supported early discharge	Point of need access	Individualised follow‐up care
Brown et al. (2002) UK [17]	✔								
Capelan et al. (2017) UK [18]		✔	✔						
Chapman et al. (2009) UK [19]				✔					
Jenkins et al. (2023) UK [20]	✔		✔						
Karlsen et al. (2023) Denmark [21]			✔						
Kirshbaum et al. (2016) UK [22]		✔							
Koinberg et al. (2004) Sweden [23]					✔	✔			
Moore et al. (2022) UK [24]	✔						✔		
Sheppard et al. (2009) UK [25]								✔	
Riis et al. (2020) Denmark [26]									✔

### 3.1 Characteristics

Of the ten studies, seven were conducted in the UK, two in Denmark and one in Sweden. There were a variety of study designs employed, as well as a range disease stage and age of patients as demonstrated in Table 1.

### 3.2 Outcome Measures

Across ten studies there were fourteen parameters used as outcome measures. The most common parameter was service use, reported by seven studies [17, 19, 21, 24, 34, 35, 36]. This was followed by the HLQ‐C30 tool used in five studies [17, 21, 34, 36, 37]. The QLQ BR23, HADS and questionnaires were employed in four studies each and the remainder of outcome measures (recurrence rates, interviews, HNA, GHQ‐12, GSE, EQ 5D‐5L and FACT‐B) were used in three or fewer studies.

### 3.3 Mechanism of Follow‐Up

All of the reported pathways included automatically scheduled mammography, but frequency of mammograms ranged from one to two yearly, and duration of surveillance before discharge to national screening was reported as 5 years [22], 3 years [38], or no end date for annual mammography was given [18, 19, 25, 34, 36, 39]. Eight out of ten [17, 18, 19, 20, 25, 35, 36, 37] reported pathways involved a meeting with a specialist nurse at the end of surgical treatment, and six out of ten [37] specified that written information was given to the patient at that appointment.

### 3.4 Common Terminology

Nine phrases were used to refer to the concept of “SDA’ pathways in the included studies. The term “Patient‐Initiated Follow Up” was used by three studies [17, 24, 34] as was “Self‐Supported Management” [18, 20, 21]. “Open Access” was used by two studies [18, 22]. The remaining six phrases appeared in one study each.

### 3.5 Recurrence

Recurrence was reported by 3 studies [17, 25, 35] all of which were randomised control trials (Table 5), and no study suggested that patients had worse cancer outcomes being managed via a patient‐led system of follow‐up.

**TABLE 5:** Recurrence outcomes.

Study	Timeframe	Control group recurrence	Local versus distant	SDA group recurrence	Local versus distant
Brown et al. 2002 [17]	12 months	2/30 (6%)		2/31 (6%)	
Koinberg et al. 2004 [23]	5 years	17/131 (13%)	Local 8/131 (6%)	17/133 (13%)	Local 12/133 (9%)
			Distant 9/131 (7%)		Distant 9/133 (7%)
Sheppard et al. 2009 [25]	18 months	4/112 (4%)	Local 1/112 (< 1%)	5/112 (4%)	Local 1/112 (< 1%)
			Distant 3/112 (2%)		Distant 4/112 (3%)

Two studies reported on the mode of detection of recurrences. Brown et al. [17] reported that both recurrences within the SDA group were referred to outpatient clinic (OPC) by their GP. Sheppard et al. [25] reported 5/9 recurrences were distant and detected as emergency presentations to hospital, with no detail given on what type of follow‐up these patients were receiving.

### 3.6 Resource Use

Six studies reported on the reduction in OPC visits with a patient‐led system of follow‐up as shown in Table 6 [17, 25, 36]. One study explored the effect of patient‐led follow‐up on the workload of GPs. In their audit Chapman et al. [19] found that only 10 out of 277 GPs had to re‐refer a patient back to the breast unit during the 3 years the programme had been running. 71 GPs reported no consultations with patients in the programme, and only 13 GPs had seen patients 3 or more times.

**TABLE 6:** Resource utilisation.

Study	Sample size	Timeframe	Standard	SDA
Brown et al. 2002 [17]	61	12 months	• 1 BCN phonecall • 3 GP referrals to OPC	• 1 BCN phonecall • 4 GP referrals to OPC
Chapman et al. 2009 [19]	130	31 months		• 5 OPC required
Jenkins et al. 2023 [20]	110	12 months		• 54% contacted GP at least once • 25% contacted practice nurse • 53% required OPC
Koinberg et al. 2004 [23]	264	5 years		• 450 fewer OPC • 177 more BCN phonecalls • 88 more BCN visits
Riis et al. 2020 [26]	134	24 months	• Twice as many OPC	
Sheppard et al. 2009 [25]	237	18 months	0.42 contacts/person year	0.38 contacts/person year

Only one study conducted a cost analysis for the SDA pathway. The observational study (PRAGMATIC by Jenkins et al. [34]) included measures of cost and resource utilisation over a 12 month period. Units of cost compared included nursing time, contact with hospital breast care providers and GP contact. No costs were provided for historic pathways of routine outpatient follow‐up for comparison. The mean cost of healthcare contact and care provided by family and friends was £627.75 per patient (95% C.I. £456.45 and £799.04). Patients also self‐reported private breast cancer related expenditure whilst on the SDA pathway, but there were no comparator data collected on patient's outlay when on the routine follow‐up pathway. Patients were invited to report if they had expenditure related to treatment travel, prescriptions, over the counter medicines, or special garments/prostheses. The mean cost incurred for these items over 12 months was £118.24.

### 3.7 Thematic Analysis

Within the ten studies there were two broad categories of themes identified—that of unmet needs of patients on PIFU pathways, and the lived experiences of these patients. These themes and their sub‐themes are shown in Supporting Information S1: Appendix 2.

#### 3.7.1 Theme 1: Unmet Needs

Patient responses highlighted several unmet needs for patients managed via Self‐Directed Aftercare pathways, principally in the categories of information provision and psychological support.

##### 3.7.1.1 Information

Unmet information needs were common for those patients managed by Self‐Directed pathways. Analysis demonstrated that information needs are often highest at diagnosis [24, 35, 38] and then in the year or 18 months after treatment has been completed but treatment‐related changes are still occurring within the breast [20, 24]. Among the reported SDA pathways, all have a consultation with a breast nurse to address perceived information needs immediately after treatment, but none offered a further ‘checkpoint’ down the line.

A consistent unmet information need was the lack of knowledge and confidence to conduct breast self‐examination. Across several studies [20, 24, 34, 35], patients reported that their breasts continued to change following treatment, making it difficult to know what were ‘normal’ or expected changes. Patients also reported reluctance or feeling unable to ask for help with self‐examination.

I've got scars and things so it's quite difficult for me to know how it should feel….I don't feel confident that I can check.

[24]

…it should maybe have been in about 18 months from [last appointment] it would have been good to see someone….

[24]

##### 3.7.1.2 Psychological

Results demonstrated a mismatch between qualitative and quantitative findings regarding psychological needs.

Surveys alone concluded that there were no objective differences demonstrated between reported anxiety and depression in patients managed via SDA versus routine outpatient review [17, 35], but unmet psychological needs were frequently reported by SDA patients in interviews [17, 24, 25, 34, 38].

Commonly, patients undergoing SDA reported feelings of isolation and abandonment [19, 20, 24]. Despite this, they did not feel they could contact their health care team, or did not feel that psychological needs were in the remit of their surgical team [20]. The lack of routine appointments reduced the opportunity for patients to discuss psychological issues [20, 24] resulting in some patients reporting out of pocket expenses to seek psychological support from other avenues [34].

Patients across studies reported high levels of anxiety linked to worries about cancer recurrence and how to self‐examine or detect the symptoms associated with recurrence. These anxiety levels remained high in patients where risk was low [20, 38].

I’m on my own. A small part of me feels that that’s not quite right. A tiny part of me feels a bit abandoned.

[20]

#### 3.7.2 Theme 2: Experience of the Service

Results identified two key subthemes regarding patients' lived experience of SDA—accessibility and reassurance.

##### 3.7.2.1 Accessibility

Some patients reported enjoying easy access to their clinical team and a positive and rapid resolution to queries [24]. Others maintain they would have been more comfortable seeing a healthcare member more regularly, or that they struggled to access the hospital point of contact [24]. Many patients reported high levels of trust and confidence in their hospital team, and positive experiences of dealing directly with Breast Care Nurses [16, 17, 18, 19, 20, 21].

Other patients did not feel as confident or empowered to contact their breast team. Analysis demonstrates that even when patients confirmed they were aware of the pathway to access support [16, 18] there was reluctance to access it due to both experienced and perceived barriers [19, 20]. Experienced barriers included slow response from breast care nurses for example voicemail messages being responded to after several days [24], or not being aware of a clear method of contacting the hospital team [24, 40]. Perceived barriers also included patients feeling their symptoms were not important enough or within the remit of the breast team for example psychological needs [19, 21].

Studies identified that convenience was important to patients accessing services. Across studies patients valued reduced travel costs and time saved with not attending a routine appointment when they had no issues to raise [20]. They appreciated receiving phone‐call advice at a time convenient to them rather than needing to attend a face‐to‐face appointment [21]. Group education events and support programmes were recognised by patients as an important component of SDA [17]. Despite this, these programmes reported poor attendance due to inconvenient timing and the need to travel to face‐to‐face events [19].

What could be a very simple question became aggravated by the days of anxiety of not getting an answer.

[19]

I’m hugely impressed with the care of the breast care nurses.

[21]

All respondents were satisfied or very satisfied with process to contact the breast unit.

[16]

…you know you are going to get a call back and I don’t need to book time off work.

[21]

##### 3.7.2.2 Reassurance

Patients reported mixed views on how reassured they felt with SDA and it was a theme common to many of the included studies. The ability to contact the healthcare team directly was felt to be reassuring by some patients who viewed it as efficient access to specialist advice or review (18, 20). Other patients reflected on the lack of reassurance from loss of scheduled contact with the hospital team and would have preferred the comfort of a scheduled visit with a doctor in the breast clinic [17, 20, 21].

It feels reassuring to know that I have the breast care nurses helpline to call if I do have any concerns.

[21]

I think for a lot of people seeing a doctor gives them confirmation.

[17]

They could have shown us more and brought women in the same situation together.

[17]

## 4 Discussion

This scoping review has identified that the literature reporting on outcomes in breast cancer SDA is sparse and of varying quality. Only ten studies were identified reporting on the impact SDA had on patients, and there was little consensus over who could be managed by SDA, what the pathway should include, or how to report on SDA outcomes. There was conflicting evidence of how accessible patients found SDA pathways, with wide variation in both experiences and perceived accessibility. No studies were powered to detect differences in survival or recurrence rates, but none showed worse cancer outcomes for patients on SDA. Included studies had a high level of heterogeneity in study design, inclusion criteria, reporting measures and mechanism and description of Self‐Directed Aftercare. This makes it difficult to determine the wider applicability of results, and agree a standard of care to be provided.

Studies in this review indicated potential cost‐savings with SDA by reducing outpatient clinic visits, but this alone cannot be used as a measure of economic success without the context of workload and training required for Breast Care Nurses and other members of the healthcare team. The cost‐benefit of intensive surveillance programmes in early breast cancer has long been a subject of interest in countries with nationalised health systems [41]. Evidence has shown that more intensive clinical reviews [42] convey no advantage for disease‐free or overall‐survival over less‐intensive pathways, but carry a more than two‐fold increase in cost. It is unclear from this review or other published evidence whether patients are financially penalised by a particular type of follow‐up. There is now a wider understanding internationally of the hidden financial toll of cancer care on patients [43] and the cost of SDA to patients merits further exploration in qualitative and health economics research.

Results demonstrated mixed experiences of SDA with some patients finding the service reassuring, echoing literature suggest that patients find the concept of SDA to be acceptable [44], whilst other patients struggled with the loss of scheduled contact with HCPs, in keeping with reported reductions in cancer‐related worries around the time of a scheduled clinical review [45]. There was no consensus in the data regarding unmet needs, and deeper analysis reveals an important mismatch between quantitative and qualitative data. Survey data were largely positive with high rates of satisfaction and no issues reported, and yet in interviews patients repeatedly reported unmet needs, high levels of anxiety and issues with contacting their health team. This demonstrates that quantitative data alone is not sufficient to evaluate patients' experience of such a complex healthcare intervention, and mixed‐methods research gives a fuller picture of lived experience and not simply service use.

No studies in this review included the perspectives of healthcare professionals (HCPs) involved in delivering SDA. Literature exploring this group [46] noted that HCPs generally viewed proposals for SDA favourably, but that the provision of this was contingent on providing accurate and accessible information to patients at the right time, having a clear pathway for contacting the hospital team, and managing the fear of cancer recurrence. Again these are useful insights into the perceived opportunities and challenges of SDA, but it is vital to seek out insights from those with lived experience of SDA, particularly when it is so widely practiced in the UK and further field.

Of note there are other examples in the literature of attempts to explore alternative systems of ‘de‐escalated hospital follow‐up’ in breast cancer, which remain healthcare‐professional led and offer patients regular review regardless of symptoms (as opposed to patient‐led symptomatic review with Self—Directed Aftercare). Some of these pathways have included telephone review by the hospital physicians [47], regular review by GP [47, 48], regular nurse‐led review [49] or even radiographer‐led review [50]. Systematic reviews [47, 48] assessing these alternative pathways have concluded that the quality and magnitude of these studies is variable and largely too scant to support meaningful conclusions; that being said it is likely that there is a cost reduction associated with all of these methods compared to regular face‐to‐face hospital follow‐up, but it is unclear what the impact of these follow‐up systems is on the quality of life or survivorship of patients, and whether they have ongoing unmet needs on these pathways.

This review has identified a critical timepoint common across studies, where many patients begin to experience and/or report unmet needs between 18 months and 2 years following treatment. There may be an opportunity to adapt current pathways to include a pre‐emptive ‘checkpoint’ around 18 months to establish what patients' current unmet needs are, and how they can best be addressed. With regards to how support is offered, results suggest that patients prioritise convenience over perceived benefits of some interventions. A co‐design approach with service users has been extensively validated for use in evaluating and designing cancer services [49, 50] and would facilitate the provision of services which are useful and accessible to patients.

There is currently a limited evidence base regarding SDA. It has been established by high quality studies in breast cancer that there is no improvement in Disease‐Free Survival (DFS) or Overall Survival (OS) with intensive follow‐up (examinations, imaging, bloods tests) compared to regular mammography alone [4, 7, 10, 51, 52]. Despite this there is no consensus or high‐quality research on what alternative, less‐intensive and patient‐initiated follow‐up should look like, as shown by the heterogeneity of studies in this review. Studies reported here have short follow‐up, lack comparison groups or are retrospective and observational in nature. None of the studies explicitly explores the experience of patients who have developed a recurrence whilst being managed via SDA, or the experiences of those managed via historic regular clinical review. None of the included studies mention the experience of patients' carers or loved ones, which we know is impactful for the patient's own quality of life as well as the carer's mental and physical health [53, 54, 55, 56, 57]

### 4.1 Clinical and Research Implications

From this review it would appear that there is a wide range of experience for patients who are managed via SDA, and that patients' needs at their time of initial assessment (immediately after surgery) may not accurately reflect their level of ongoing need. It is clear that many women may be superficially aware of the mode of follow‐up, but many are still not confident with how or when to contact the hospital team, or are reluctant to do so. Many patients require educational messages or pathways to be reiterated at different times in their journey, which is currently not being provided.

For psycho‐oncology research, this work highlights the need for further high‐quality mix‐methods research exploring:

- the experiences of women who have been managed via SDA for longer periods of time, including those who developed recurrence on SDA
- the experiences of caregivers or those close to breast cancer patients managed via SDA
- the experiences of healthcare staff delivering SDA
- targeted health economic evaluation to establish the resource consumption of SDA

### 4.2 Limitations of This Review

(1) This review only considered primary articles and as such may have overlooked useful insights from related systematic reviews or articles considering cancer follow‐up from multiple sites where outcome data is merged. (2) The search strategy was developed iteratively to include as many key terms and combinations as possible that were relevant to Self‐Directed Aftercare pathways, however there may been less commonly used terminologies for such pathways that have not been captured in our review. Within individual studies more than one phrase was often used to refer to Self‐Directed Aftercare pathways. (3) Although the quality of the included studies has been commented on in this review, we have not formally evaluated quality nor made it an exclusion criterion; largely, this is due to the small volume of literature available on the subject matter.

## 5 Conclusions

Despite the limited evidence available as shown in this review, SDA continues to be widely employed by hospitals across the UK in efforts to meet NICE guidelines and rationalise resources in a way that meets patients' needs. This remains the case for other countries in Europe, as demonstrated by the geographical spread of studies in this review.

It is important for research to engage with other stakeholders that is clinical staff and carers/loved ones of patients. As there seems to be a trend for changing needs emerging around 18–24 months, future research could incorporate trialling a ‘checkpoint’ for patients at this stage to anticipate developing issues.

This work has highlighted the potential discordance between quantitative and qualitative feedback from patients. It is clear from qualitative data in these studies that there remain an overwhelming number of unmet needs for patients at different timepoints, and that there is a wide range of user experience when being managed via an SDA pathway. Further high‐quality mixed‐methods research is needed to assess the outcomes and lived experiences of patients, their carers, and staff involved in delivering these pathways.

## Author Contributions

This work was performed as part of a doctoral research programme by AI, supervised by OS, GI and SMcI. These authors conceptualised the project and acquired doctoral funding for AI. Data curation and validation were conducted by AI, KM and LC. OS, AI and SMcI completed analysis of data according to appropriate methodology. The first draft of the manuscript was written by Anna Isaac and all authors read and commented on subsequent versions of the manuscript. All authors read and approved the final manuscript.

## Funding

This work was supported by a research doctoral fellow grant from the Public Health Agency for author A.I. Grant number: EAT/5741/22.

## Conflicts of Interest

The authors declare no conflicts of interest.

## Supporting information


Supporting Information S1

## Data Availability

The data that support the findings of this study are available in PubMed at https://pubmed.ncbi.nlm.nih.gov. These data were derived from the following resources available in the public domain: ‐ Web of Science, https://www.webofscience.com/wos/woscc/smart‐search ‐ OVID Medline, https://ovidsp‐dc1‐ovid‐com.qub.idm.oclc.org/ovid‐new‐a/ovidweb ‐ PubMed, https://pubmed.ncbi.nlm.nih.gov.
