# Supplementary material for: The Impact of Self‐Directed Aftercare Following Breast Cancer Surgery: A Scoping Review
Source: Psychooncology. 2025 Dec 29;35(1):e70368. doi: 10.1002/pon.70368 (PMC12747846; doi:10.1002/pon.70368)
Supplement: Supplementary file 1 — Supporting Information S1 [file PON-35-e70368-s001.docx]

Appendices

**Appendix 1**

Search terms

| **Topic** | **Search terms** |
| --- | --- |
| Breast cancer | Breast cancer |
| Patient-initiated | Patient-led  OR  Self-directed  OR  Patient-directed  OR  Self-led  OR  Self-initiated  OR  Open access |
| Follow-up | After-care  OR  Follow-up |

Search strategy used for Web of Science

| **Key term** | **Key term** | **Key term** | **Results (title screened)** |
| --- | --- | --- | --- |
| Breast cancer | Patient-directed | Aftercare | 0 |
| Breast cancer | Patient-directed | Follow-up | 0 |
| Breast cancer | Patient-initiated | aftercare | 0 (1) |
| Breast cancer | Patient-initiated | Follow-up | 21 (78) |
| Breast cancer | Patient-led | Aftercare | 0 |
| Breast cancer | Patient-led | Follow-up | 13 (34) |
| Breast cancer | Self-directed | aftercare | 2 |
| Breast cancer | Self-directed | Follow-up | 4 (19) |
| Breast cancer | Self-initiated | Aftercare | 0 |
| Breast cancer | Self-initiated | Follow-up | 0 (1) |
| Breast cancer | Self-led | Aftercare | 0 |
| Breast cancer | Self-led | Follow-up | 0 |
| Breast cancer | Open access | Follow-up | 11 (621) |
| Breast cancer | Open access | aftercare | 1 (5) |

Search strategy used for PubMed

| **Key term** | **Key term** | **Key term** | **Results (title screened)** |
| --- | --- | --- | --- |
| Breast cancer | Patient-directed | Aftercare | 0 (0) |
| Breast cancer | Patient-directed | Follow-up | 0 (3) not PIFU |
| Breast cancer | Patient-initiated | aftercare | 1 (1) |
| Breast cancer | Patient-initiated | Follow-up | 10 (21)  6 systematic reviews  5 not PIFU |
| Breast cancer | Patient-led | Aftercare | 2 (6) 2 reviews  2 not PIFU |
| Breast cancer | Patient-led | Follow-up | 4 (6) |
| Breast cancer | Self-directed | aftercare | 0 (6) |
| Breast cancer | Self-directed | Follow-up | 0 (11) not PIFU |
| Breast cancer | Self-initiated | Aftercare | 0 |
| Breast cancer | Self-initiated | Follow-up | 0 |
| Breast cancer | Self-led | Aftercare | 0 |
| Breast cancer | Self-led | Follow-up | 0 |
| Breast cancer | Open access | Aftercare | 2 (6) not PIFU |
| Breast cancer | Open access | Follow-up | 6 (71) 3 review  62 not PIFU |

**Search strategy used for MEDLINE**

|  | [# ▼](https://ovidsp.dc1.ovid.com/ovid-new-a/ovidweb.cgi?&S=MOIDFPIMNAACMDKDKPJJGGPMDGIDAA00&Sort+Sets=ascending) |  |
| --- | --- | --- |
|  | | |
|  | 13 | 1 and 11 and 12 |
|  | 12 | 3 or 7 |
|  | 11 | 2 or 4 or 5 or 6 or 8 or 9 or 10 |
|  | 10 | patient-led.mp. |
|  | 9 | patient-directed.mp. |
|  | 8 | patient-initiated.mp. |
|  | 7 | aftercare.mp. or Aftercare/ |
|  | 6 | self-initiated.mp. |
|  | 5 | self-led.mp. |
|  | 4 | self-directed.mp. |
|  | 3 | follow-up.mp. |
|  | 2 | open access.mp. |
|  | 1 | breast cancer.mp. or Breast Neoplasms/ |
|  | | |

***Appendix 2***

***Themes***

| **Themes** | **Supporting results** |
| --- | --- |
| **Unmet needs** |  |
|  | *"They could have shown us more and brought women in the same situation together and described in more detail this is how things are and this is what we're going to do….A bit more detailed information could have been good…"* [[33](#_ENREF_33)] |
|  | *" I still have unanswered questions, but that's more important in the beginning because you have more questions then"* [[37](#_ENREF_37)] |
|  | *"What they don’t then say is actually it's going to impact on your sex life and impact on your relationship. Nobody had that discussion with me.”* [[23](#_ENREF_23)] |
|  | *"I felt the follow-up [HNA] was far too late after the end of treatment. Because all my sort of concerns or worries I'd addressed myself at that point, or the majority of them"* [[23](#_ENREF_23)] |
|  | “Specific concerns included ease of access to additional psychological support or advice about treatment side effects including post-menopausal symptoms.” [[18](#_ENREF_18)] |
|  | *"You can examine yourself but you just need somebody to confirm and say yes you haven’t found anything”. [*[*16*](#_ENREF_16)*]* |
| Information | *"No they didn't really tell you how to check [breast self-examination]. I think they kind of expected you to know how to check…"* [[23](#_ENREF_23)] |
|  | *"It's no good saying just get used to your new normal, because there is no normal, it keeps changing” [*[*23*](#_ENREF_23)*]* |
|  | *"Not very confident, as I've had scarring….worry I wouldn't notice any changes as the scar tissue feels like a lump"[*[*19*](#_ENREF_19)*]* |
|  | *"I've got scars and things so it's quite difficult for me to know how it should feel….I don't feel confident that I can check and know what my body should feel like, in terms of self-examination"[*[*23*](#_ENREF_23)*]*“ |
|  | Male patients: *"…understandably a lot of the literature is written for women….it's hard to read about the other gender and it feels like you don't fit in"* [[19](#_ENREF_19)]*"* |

|  |  |
| --- | --- |
|  | Drivers of total out of pocket costs included "experiencing psychological morbidity" [[36](#_ENREF_36)] |
|  | *"…having availability of psychological support, a year, 18 months down the line…that can possibly be the time that really you need the psychological support more"* [[23](#_ENREF_23)] |
|  | *"Once you're discharged you don't sort of have any backup for potential recurrence, and I almost feel out on a limb….it's that void afterwards, that is my only criticism, because it's almost like a loaded gun and you're waiting for somebody to fire the bullet."[*[*23*](#_ENREF_23)*]* |
|  | *“Because you have had breast cancer you are afraid of pressing too hard in case you find something”[*[*16*](#_ENREF_16)*]* |
|  | *"I'm constantly tormented by the fear that it will spread to my other breast"* [[33](#_ENREF_33)] |
| Psychological | *…wouldn't go to them [* *BCNs] with psychologic* *al problems - it's not their remit really"* [[19](#_ENREF_19)] |
|  | Comparing PIFU with standard outpatient follow-up: "there were no statistically significant differences regarding anxiety and depression between the groups"[[37](#_ENREF_37)] |
|  | “Women treated for early breast cancer were not disadvantaged by allocation to open access…in terms of quality of life experienced" [[21](#_ENREF_21)] |
|  | *"I do think about [recurrence] daily….I would say I'm frightened the cancer's going to pop out somewhere else. I don't feel quite in control of it…"* [[23](#_ENREF_23)] |
|  | *"…you can't help but worry if it spreads somewhere else and you won't necessarily know." [*[*19*](#_ENREF_19)*]* |

| **Lived experience of PIFU** |  |
| --- | --- |
|  | *"…would go to the BCN helpline first, then the oncologist. Not my GP because they are the middle-man"[*[*19*](#_ENREF_19)*]* |
|  | *"I'm hugely impressed with the care of the breast care nurses…They are my first port of call for any breast cancer related concerns, it's their area of specialty. I'd always speak to them first rather than my GP."[*[*19*](#_ENREF_19)*]* |
|  | *"They [BCNs] are experts in the breast cancer field it is more reassuring [to go to the BCNs] than going to the doctor [GP]"[*[*19*](#_ENREF_19)*]* |
|  | *"I feel trust in the nurse as well as the doctor"* [[33](#_ENREF_33)] |
|  | *"They [nurse helpline] were brilliant when I rang them. And they saw me quickly and followed me up...I am reassured that if I am concerned they're there and they will see me and they will fit me in as quickly as they can"* [[23](#_ENREF_23)] *"* |
|  | *"I could phone the same nurse and talk to her and if I was especially worried, like in the beginning, then she arranged an appointment with the doctor so it went very smoothly"* [[33](#_ENREF_33)]*"* |
|  | “Both women [with recurrence] in the patient initiated group were referred to the hospital by their GPs” [[16](#_ENREF_16)] |
| Accessibility | "All respondents satisfied or very satisfied with process to contact the breast unit" [[18](#_ENREF_18)] |
|  | “Patient satisfaction with access to the medical centre was ranked as high over the follow-up period ranging from 93% to 100%.” [[37](#_ENREF_37)] |
|  | *"it feels like you can't call the nurses once you've been discharged, you're just like you're discharged, now you're back at the GP, go and see them"* [[23](#_ENREF_23)] |
|  | *“It would be really handy to have an email address or even a number you could text, not expecting an instant answer…because by the time you've rung two or three times and they’ve not picked up and you don't really want to leave a message you get to the point where you think maybe it's not that important and I won't ring again....so it's more an access issue, in that....you don't want to be a problem and they are obviously very busy"[*[*23*](#_ENREF_23)*]* |
|  | *"I did know something was wrong and kept ringing [the hospital helpline] but it took them quite a while to actually listen to me"* [[23](#_ENREF_23)] |
|  | *"Those experiences [of calling the hospital helpline] that I found aggravated my anxiety, so I would be more likely to try to speak to my GP rather than leave a message on an answering machine for someone who…might not call back for a few days…what could be a very simple question became aggravated by the days of anxiety of not getting an answer"[*[*23*](#_ENREF_23)*]"* |
|  | “*I think for a lot of people seeing a doctor gives them confirmation”[*[*33*](#_ENREF_33)*]* |
|  | Of PIFU system: *"it gives you…peace of mind you know, that they are still keeping an eye on you"[*[*16*](#_ENREF_16)*]* |
|  | *“I’m on my own. A small part of me feels that that’s not quite right. A tiny part of me feels a bit abandoned”* [[19](#_ENREF_19)] |
|  | *“It seems a bit odd not to have a single consultation…”[*[*19*](#_ENREF_19)*]*] |
|  | *“At the beginning I felt abandoned…[*[*19*](#_ENREF_19)*]* |
|  | *"Even if it was just a 20 minute appointment to bring out any things that you don't want to go to the doctors [GP] to talk about…it should maybe have been in about 18 months from [last appointment] it would have been good to see someone"* *[*[*23*](#_ENREF_23)*]* |
|  | *"Just little comments could have helped to make it feel like we're still here, we're always here…And I know that is open to me, so that if I felt concerned or worried I could ring [the hospital helpline] and I could go on in, but it just didn't feel like it on the day [of HNA]"[*[*23*](#_ENREF_23)*]* |
|  | *"Once you're discharged you don't sort of have any backup for potential recurrence, and I almost feel out on a limb….it's that void afterwards, that is my only criticism, because it's almost like a loaded gun and you're waiting for somebody to fire the bullet."[*[*23*](#_ENREF_23)*]* |

|  |  |
| --- | --- |
|  | *"They [nurse helpline] were brilliant when I rang them. And they saw me quickly and followed me up...I am reassured that if I am concerned they're there and they will see me and they will fit me in as quickly as they can"* [[23](#_ENREF_23)] |
|  | *"…one wants to hear every day that one hasn't got cancer and that's not possible but that's about how I feel."[*[*33*](#_ENREF_33)*]* |
| Reassurance | *"It feels reassuring to know that I have the breast care nurses helpline to call if I do have any concerns"*[[19](#_ENREF_19)] |
|  | Of PIFU system: *"it's just nice to know that number's there for support if I do find anything"*[[23](#_ENREF_23)] |
|  | *“I used to dread going…not because I was worried about what the outcome would be, but because it was a heck of a journey from here to Southampton and the waiting around etc”[*[*16*](#_ENREF_16)*]* |
|  | *“…you know you are going to get a call back and I don’t need to book time off work*”[[19](#_ENREF_19)] |
| Convenience | Most women recalled being signposted to Living Well support programs; however uptake was very low…Barriers to attendance included….practical barriers of distance [or] being busy [[23](#_ENREF_23)] |
|  | *"I just wanted to get it done and dusted and out of there. I don't want to be followed up really. I don't want to be reminded that it happened.” [*[*23*](#_ENREF_23)*]* |
| Autonomy | *"It's taking back control of your own body, knowing what to look out for"[*[*19*](#_ENREF_19)*]* |
|  | *"I do think about [recurrence] daily….I would say I'm frightened the cancer's going to pop out somewhere else. I don't feel quite in control of it…"* [[23](#_ENREF_23)] |
|  | *“It's taking back control of your own body, knowing what to look out for” [*[*19*](#_ENREF_19)*]* |
|  | *"Up to me now to get on with it and come to terms with it…..It should help me stop worrying about every ache and pain"[*[*19*](#_ENREF_19)*]* |
